# Robust Time-Delay Feedback Control of Vehicular CACC Systems with Uncertain Dynamics

**DOI:** 10.3390/s20061775

**Published:** 2020-03-23

**Authors:** Xiulan Song, Li Chen, Ke Wang, Defeng He

**Affiliations:** College of Information Engineering, Zhejiang University of Technology, Hangzhou 310023, China; 201603090402@zjut.edu.cn (L.C.); 1416627273@zjut.edu.cn (K.W.); hdfzj@zjut.edu.cn (D.H.)

**Keywords:** cooperative adaptive cruise control, vehicle platoons, networked control systems, robust stability, string stability

## Abstract

This paper proposes a new, robust time-delay cooperative adaptive cruise control (CACC) approach for vehicle platooning systems with uncertain dynamics and varying communication delay. The uncertain CACC models with perturbed parameters are used to describe the uncertain dynamics of the vehicle platooning system. By combining the constant time headway strategy and predecessor-following communication topology, a set of robust delay feedback controllers is designed for the uncertain vehicle platoon with varying communication delay. Then, the set of CACC controllers is computed by solving some linear matrix inequalities, which further establish the robust (string) stability of the uncertain platooning system with the varying communication delay. The co-simulation experiment of CarSim and Simulink with a group of a seven-car platoons and varying velocity is used to demonstrate the effectiveness of the presented method.

## 1. Introduction

Recently, cooperative adaptive cruise control of a group of connected and automated vehicles (CAVs) has attracted considerable attention in both industrial and academic communities. With the information exchange among vehicles in the group by vehicle-to-anything (V2X) communication [1], CACC systems can improve traffic flow stability, throughput, driving safety and ride comfort of vehicles [2,3]. CACC technology is now shown to be one of the promising intelligent transportation system technologies [2,3,4,5,6,7,8,9].

The main objective of CACC is to adjust the group of CAVs to form a vehicle platoon with keeping a desired safe inter-vehicle distance, i.e., spacing. Besides the stability issue of CACC systems, the string stability of vehicle platoons [2,3] is the key aim of theoretical analysis of the vehicular CACC system. The string stability property of vehicle platoons implies that the perturbation from the leading vehicle of a platoon will not be amplified downstream through the platoon [2,3]. This property can effectively avoid a halt or collision at the end of a platoon [10,11]. Various CACC methods have been proposed to derive the (string) stability of vehicle platoons (e.g., see [5,6,7,8,9,12,13,14,15,16,17,18] and the references therein).

In CACC systems, the real-time behaviors of front vehicles are dispatched to the nearest following vehicles by the V2X wireless network. Hence, the V2X network has important effects on the (string) stability of vehicular CACC systems due to varying communication delays [3]. Many efforts addressed these effects within the networked control systems framework [19,20,21,22,23,24,25]. For instance, the effect of network-induced constant delays or sample-and-hold devices on string stability was studied within a networked control system perspective [19]. By using the Smith predictor [26], which is known to be robust against time-delay, a model-based robust *H*_∞_ control was presented for a granulation process with reference updating [27]. The most results on CACC were derived by assuming constant time-delay of the V2X network and few results have been obtained to deal with varying heterogeneous communication delay. For instance, the author in [24] proposed a distributed consensus strategy for control of the vehicle platoon represented by second order integral systems with varying heterogeneous communication delay. In the previous work [25], we designed CACC controllers for nominal vehicle platoon systems, i.e., the CACC systems are assumed to be modelled exactly. However, in actual it is hard to derive an exactly CACC model for a real CACC system. Moreover, the unknown dynamics of a CACC system will degrade the controllers based on the nominal CACC system. Here the CACC controllers in [25] will be generalized as robust CACC controllers by taking into account the uncertain dynamics of CACC systems.

In most of the existing CACC methods, the dynamic models and parameters of systems are assumed be partially or fully known for design of CACC controllers. The assumption may degrade closed-loop performance in the face of parametric uncertainties and/or unknown disturbances. Compared to a lot of nominal CACC results, fewer work considered the model uncertainties in CACC systems. For example, in [28], an adaptive optimal control strategy was proposed for heterogeneous CACC system with uncertain dynamics by resorting adaptive dynamic programming. The authors in [29] presented a robust distributed control protocols for vehicle platoons with unknown nonlinear dynamics under both the predecessor-following and the bidirectional control configurations. Considering parametric uncertainties and uniform communication delay, in [30], the robust *H*_∞_ control method was introduced for vehicle platoons with fully networked bidirectional control structure and the constant distance headway strategy. It remains challenging to understand how the combination of uncertain dynamics and heterogeneous communication delay affects the cooperative cruise control of vehicle platoons.

The aim of this paper is to solve robust CACC problems in the networked control framework by taking into account parametric uncertainties and heterogeneous communication delay. By adopting the constant time headway strategy and predecessor-following communication topology, the vehicular CACC systems with uncertain dynamics and heterogeneous communication delay are modeled as a family of uncertain car-following models with perturbed parameters and varying time-delay. Then, a set of robust delay feedback controllers is designed for the uncertain vehicle platoon with varying communication delay and is computed in such way that each vehicle evaluates its own controller using only neighborhood information. Moreover, the platoon formation and its stability are guaranteed in the presence of parametric uncertainties and varying time-delay by some linear matrix inequalities (LMI) conditions. With respect to the constant distance headway strategy, here, the car-following models consider the constant time headway which enables a vehicle to proportionally adjust to the desired spacing based on its velocity [2,3,4]. Hence, the used models are more appropriate in the real-world applications. Finally, numerical results of co-simulation experiments of CarSim and Simulink illustrate the effectiveness of the proposed CACC method for string stability of a seven-vehicle platoon in the varying speed scenario.

Notations: Throughout the paper, *P* > 0 means that the matrix *P* is positive definite and diag{...} denotes a block-diagonal matrix. The symbol *I* denotes the identity matrix with compatible dimensions and traditional symbol ‘T’ denotes the transpose of a vector or a matrix. The symmetric terms in a symmetric matrix are denoted by ‘*’, e.g., $\left[\begin{array}{cc} M & N\\ {}* & L \end{array}\right]=\left[\begin{array}{cc} M & N\\ N^{T} & L \end{array}\right]$.

## 2. Problem Formulation

**Figure 1:**
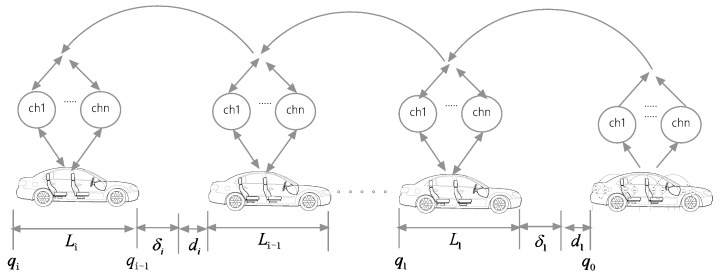
A schematic CACC system of the vehicle platoon.

Consider a group string of *N* + 1 vehicles moving along a single lane and assume that they run in horizontal environment (see Figure 1), where the first vehicle in the platoon presents the reference trajectory of the string according to some safety spacing policies. In this platoon, each vehicle shares its acceleration with the following vehicles through a predecessor-following (PF) communication network consisting of multiple redundant channels. Due to using multi-channel transmission, here we assume that the network has no packet loss but may exist communication delay for CACC of the vehicle platoon. The vehicles are also equipped with onboard sensors to measure the inter-vehicle distance and relative velocity with respect to its preceding vehicle. 

Consider the vehicle platoon in Figure 1 and let *L_i_*, *q_i_*, *v_i_* and *a_i_* be the length, position, velocity and acceleration of the *i*th vehicle in the platoon for *i* = 0,1,…,*N*, where *i* = 0 stands for the leading vehicle. In this figure, *δ_i_* is the spacing error and ch*j* stands for the *j*th wireless channel for *j* = 1,…,*n*. For the *i*th vehicle in the platoon, the longitudinal dynamics can be described by [7,17]

(1) $${\dot{q}}_{i}(t)=v_{i}(t),\;{\dot{v}}_{i}(t)=a_{i}(t),\;{\dot{a}}_{i}(t)=f_{i}(v_{i}(t),a_{i}(t))+g_{i}(v_{i}(t))\vartheta_{i}(t),\;\forall t\geq 0$$

where *ϑ_i_*(*t*) is the engine input of the *i*th vehicle at time *t* ≥ 0, and functions *f_i_* and *g_i_* are given by

$$\begin{array}{l} f_{i}\left(v_{i},a_{i}\right)=-\frac{1}{\varsigma_{i}}\left(a_{i}+\frac{\sigma Y_{i}f_{di}}{2m_{i}}v_{i}^{2}+\frac{p_{mi}}{m_{i}}\right)-\frac{\sigma Y_{i}f_{di}v_{i}a_{i}}{m_{i}}\\ g_{i}\left(v_{i}\right)=\frac{1}{\varsigma_{i}m_{i}} \end{array}$$

where *ς_i_* is the internal actuator dynamics of the vehicle in tracking any desired acceleration command, *σ* is the air density, and *Y_i_*, *f_di_*, *p_mi_* and *m_i_* are the cross-sectional area, drag coefficient, mechanical drag and mass of the vehicle, respectively. In order to linearize the acceleration equation in Equation (1), the following equation is used [17]:

(2) $$\vartheta_{i}=u_{i}m_{i}+\sigma Y_{i}f_{di}v_{i}^{2}/2+p_{mi}+\zeta_{i}\sigma Y_{i}f_{di}v_{i}a_{i}$$

where the new control *u_i_* is the desired acceleration command of vehicle *i*. Substituting Equation (2) into the third equation in Equation (1), we obtain a three-order linearized model that is used to represent the longitudinal dynamics of the vehicle [19], i.e.,

(3) $${\dot{q}}_{i}(t)=v_{i}(t),\;{\dot{v}}_{i}(t)=a_{i}(t),\;{\dot{a}}_{i}(t)=-1/\varsigma_{i}a_{i}+1/\varsigma_{i}u_{i}.$$

The model simplifies the complexity of modeling the longitudinal dynamics of vehicles. It has been shown that the linear model (1) adequately describes the longitudinal dynamics of the acceleration- controlled vehicles via the experimental validation and has been widely used to design CACC controllers of various vehicle platoons [19].

Note that the linear model equation (3) can be viewed as the upper layer cruise control systems in the double layers hierarchical control structure used widely by CACC systems, e.g., [7,10,19]. In this control structure, the nonlinear equations in Equation (2) are seen as the lower layer controllers of vehicles and are generally assumed to be perfectly designed in CACC systems although the aerodynamics drag force (0.5*σY_i_f_di_v*^2^) is usually hard to estimate perfectly due to unknown coefficients of drag [17,21]. In this paper, we focus ourselves on the upper layer cruise control systems in Equation (3) by assuming the desired control performance of the lower layer controllers of vehicles. 

Let the actual inter-vehicle distance of vehicle *i* be *d_i_* = *q_i_*_−1_ − *q_i_* − *L_i_*, where *q_i_*_−1_ is the position of its predecessor. In order to regulate the inter-vehicle distance *d_i_* to a small desired spacing, the constant time headway strategy is adopted as the safe spacing policy of the proposed CACC system, i.e.,

(4) $$d_{r,i}(t)=D_{i}+T_{i}v_{i}(t)$$

where *d_r_*_,*i*_ is the desired spacing between vehicles *i* and *i* − 1, *D_i_* is the desired safety inter-vehicle distance at standstill, *v_i_* is the velocity and *T_i_* is the time gap of vehicle *i*. The difference between actual and desired inter-vehicle distances defines the car-following error of each vehicle *i* as *e_i_* = *d_i_* − *d_r_*_,*I*_ = *q_i_*_−1_− *q_i_* − *L_i_* − *d_r_*_,*i*_. In this study, the vehicle platoon moves with a referred varying velocity, namely, the leading vehicle satisfies that $a_{0}(t)={\dot{v}}_{0}(t)\neq 0$.

In order to model the longitudinal CACC system of the vehicle platoon in Figure 1, the error state vector of the *i*th vehicle is selected as *x_i_* = [*e_i_* Δ*v_i_ a_i_*]^T^, where Δ*v_i_* is the relative velocity between the *i*th-pair adjacent vehicles. Then from Equations (3) and (4), the dynamics of the error variables for the vehicle can be represented as

(5) $${\dot{x}}_{i}(t)=(A_{i}+\Delta A_{i}(t))x_{i}(t)+(B_{i}+\Delta B_{i}(t))u_{i}(t)+G_{i}x_{i-1}(t)$$

where matrices

$$A_{i}=\left[\begin{array}{ccc} 0 & 1 & -T_{i}\\ 0 & 0 & -1\\ 0 & 0 & -1/\varsigma_{i} \end{array}\right],\;B_{i}=\left[\begin{array}{c} 0\\ 0\\ 1/\varsigma_{i} \end{array}\right],\;G_{i}=\left[\begin{array}{ccc} 0 & 0 & 0\\ 0 & 0 & 1\\ 0 & 0 & 0 \end{array}\right]$$

for *i* = 1,…,*N* and uncertainties Δ*A_i_* and Δ*B_i_* are the unknown parameter perturbations of the model. Note that these parameter perturbations may be caused by uncertain internal dynamics of vehicles and/or uncertain driving conditions [17,21]. Here, matrices Δ*A_i_* and Δ*B_i_* are real-valued matrix functions and can represent time-varying parameter uncertainties of the CACC system of the vehicle platoon. Moreover, the time-varying parameter uncertainties satisfy structural uncertainties

(6) $$\left[\begin{array}{cc} \Delta A_{i}(t) & \Delta B_{i} \end{array}(t)\right]=D_{i}F_{i}(t)\left[\begin{array}{cc} E_{i,1} & E_{i,2} \end{array}\right]$$

for all time *t* ≥ 0, where *D_i_*, *E_i_*_,1_, and *E_i_*_,2_ are constant matrices with appropriate dimensions and *F_i_*(*t*) is an unknown matrix function with associated dimension and satisfies that [17]

(7) $$F_{i}{}^{T}(t)F_{i}(t)\leq I,\;\forall t\geq 0.$$

Then, by using Equation (7), the uncertainties Δ*A_i_* and Δ*B_i_* are described by a set of unknown matrix functions *F_i_*. Since the *i*th vehicle receives the signals transmitted by onboard sensors and V2X communication, respectively, the output of the vehicle in the platoon is defined as

(8) $$y_{i}(t)={\left[\begin{array}{cc} x_{i}^{T}(t) & a_{i-1}(t-\tau_{i}(t)) \end{array}\right]}^{T}$$

where *τ_i_* is the unavoidable varying communication delay. Typically, *τ_i_* is ranged over some finite time intervals and can be assumed bounded by some a maximum value, i.e., 0 ≤*τ_I_* ≤*h* with the upper bound *h* > 0. In dedicated short-range communication (DSRC), the magnitude of *h* is reported as 0.1–0.4 s [21].

In [25], we proposed a time-delay feedback CACC approach for nominal vehicle platoon systems, i.e., the longitudinal CACC system in Equation (5) is free to be subject to uncertainties Δ*A_i_* and Δ*B_i_*. However, in practical there always exit some unknown uncertainties of vehicle platoon systems due to e.g. uncertain internal dynamics of vehicles and/or uncertain driving conditions [17,21]. Hence, the goal of this paper is to design CACC controllers for the system in Equation (5) such that the controllers regulate the inter-vehicle distance *d_i_* to the desired safe spacing *d_r_* as well as maintaining string stability of the vehicle platoon in the presence of some unknown uncertainties and varying delays. To this end, the robust time-delay feedback CACC controller for the *i*th vehicle in the platoon

(9) $$u_{i}(t)=K_{i}y_{i}(t)=k_{i,1}e_{i}(t)+k_{i,2}\Delta v_{i}(t)+k_{i,3}a_{i}(t)+k_{i,4}a_{i-1}(t-\tau_{i}(t))$$

is designed such that the state *x_i_* of uncertain system in Equation (5) is regulated to zero in the presence of uncertainties in Equation (6) and varying delays *τ_i_*(*t*), as well as guaranteeing string stability of the uncertain vehicle platoon in Equation (5), where the gain vector *K_i_* = [*k_i_*_,1_, *k_i_*_,2_, *k_i_*_,3_, *k_i_*_,4_] will be determined in the next section. In what follows, some concepts are defined before the design of the CACC controller.

**Definition** **1.** (Robust stability). *The uncertain CACC system in Equation (5)–Equation (6) with Equation (9) is robust stable if for a step change of referred speed at time t = 0, the system in Equation (5) is stabilized by the CACC controller in Equation (9) to the origin in the presence of uncertainties in Equation (6)*.

**Definition** **2.** (Robust string stability). *A vehicle platoon with the uncertain CACC system in Equation(5)–Equation(6) with Equation(9) is robust string stable if the CACC system is robust stable and the inequality ‖G_i_(jw)‖ ≤ 1 holds for any w ≥ 0, where G_i_(s) = a_i_(s)/a_i−1_(s), and a_i_(s) and a_i−1_(s) are the Laplace transforms of a_i_(t) and a_i−1_(t), respectively*.

Note that robust string stability is an extension of string stability from nominal to uncertain platooning systems. String stability of a vehicle platoon implies that the oscillations do not amplify downstream along the platoon, which are resulted from any maneuver of leading vehicle. Hence, string stability can be seen as a measurement on the amplification of perturbations along the vehicle string. When the onboard sensors and wireless V2X work in a normal situation, the vehicle platoon with a CACC system is string stable under some well accepted conditions, e.g., [2,3,11]. However, due to the uncertainties and the varying communication delay, the vehicle platoon with a CACC system is getting to an uncertain system subject to varying time-delay. This makes the robust CACC problem rather challenging.

## 3. Robust CACC of Vehicle Platoons

In this section, the robust controller in Equation (9) is designed to achieve the robust stability of the system in Equation (5) in the presence of uncertainties in Equation (6) and varying communication delay.

### 3.1. Closed-Loop Uncertain CACC Systems

The closed-loop CACC system is obtained by combining the dynamics of error states in Equation (5) with the CACC controller in Equation (9). To this end, this CACC controller is firstly rewritten as

(10) $$\begin{array}{l} u_{i}(t)=K_{i}{\left[\begin{array}{cc} x_{i}^{T}(t) & a_{i-1}(t-\tau_{i}(t)) \end{array}\right]}^{T}\\ \;\;\underline{\underline{\Delta }}\;K_{i,1}x_{i}(t)+K_{i,2}\begin{array}{ccc} {}[0 & 0 & 1 \end{array}]x_{i-1}(t-\tau_{i}(t)) \end{array}$$

where *K_i_*_,1_ = [*k_i_*_,1_ *k_i_*_,2_ *k_i_*_,3_] and *K_i_*_,2_ = *k_i_*_,4_, which will be computed elaborately in Section 3.2, the symbol “$\underline{\underline{\Delta }}$” represents "defined as". Then applying the CACC controller in Equation (10) into the system in Equation (5), we have the closed-loop uncertain CACC system of the *i*th vehicle

(11) $$\begin{array}{l} {\dot{x}}_{i}(t)=(A_{i}+\Delta A_{i}+B_{i}K_{i,1}+\Delta B_{i}K_{i,1})x_{i}(t)+G_{i}x_{i-1}(t)\\ \;\;\;\;+(B_{i}+\Delta B_{i})K_{i,2}\left[\begin{array}{ccc} 0 & 0 & 1 \end{array}\right]x_{i-1}(t-\tau_{i}(t)) \end{array}$$

Let *x* = [*x*_1_^T^ *x*_2_^T^⋯⋯*x_N_*^T^]^T^. From Equation (11), the closed-loop uncertain CACC system of the whole vehicle platoon can be re-written in a compact form of

(12) $$\begin{array}{ll} \dot{x}(t)= & \left(A+\Delta A+BK_{1}+\Delta BK_{1})x(t\right)\\ & +(\bar{B}+\Delta \bar{B})K_{2}Y_{1}x(t-\tau (t))\\ & +Gx_{0}(t)+{\bar{B}}_{1}K_{1,2}\left[\begin{array}{ccc} 0 & 0 & 1 \end{array}\right]x_{0}(t-\tau_{1}) \end{array}$$

with matrices

$$A=\left[\begin{array}{cccc} A_{1} & 0 & \cdots & 0\\ G_{2} & A_{2} & \cdots & 0\\ \cdots & \ddots & \ddots & \cdots \\ 0 & \cdots & G_{N} & A_{N} \end{array}\right],\;\;\bar{B}=\left[\begin{array}{cccc} 0 & 0 & \cdots & 0\\ B_{2} & 0 & \cdots & 0\\ \cdots & \ddots & \ddots & \cdots \\ 0 & \cdots & B_{N} & 0 \end{array}\right],\;\;\Delta \bar{B}=\left[\begin{array}{cccc} 0 & 0 & \cdots & 0\\ \Delta B_{2} & 0 & \cdots & 0\\ \cdots & \ddots & \ddots & \cdots \\ 0 & \cdots & \Delta B_{N} & 0 \end{array}\right],$$

Δ*A* = diag{Δ*A*_1_,Δ*A*_2_,…,Δ*A_N_*}, *B* = diag{*B*_1_,*B*_2_,…,*B_N_*}, Δ*B* = diag{Δ*B*_1_,Δ*B*_2_,…,Δ*B_N_*}, *K*_1_ = diag{*K*_1,1_, *K*_2,1_,…,*K_N_*_,1_}, *K*_2_ = diag{*K*_2,2_,*K*_3,2_,…,*K_N_*_,2_,0}, *Y*_1_ = diag{[0 0 1]_2_, [0 0 1]_3_,…,[0 0 1]*_N_*, 0}, *G* = [*G*_1_^T^,0,…,0]^T^, ${\bar{B}}_{1}=$ [*B*_1_^T^+Δ*B*_1_^T^,0,…,0]^T^ and *τ* = {*τ*_1_,*τ*_2_,..,*τ_N_*}. Moreover, substituting Equation (6) into Equation (12), it is obtained that

(13) $$\begin{array}{ll} \dot{x}(t)= & \left(A+DF(t)E_{1}+BK_{1}+DF(t)E_{2}K_{1})x(t\right)\\ & +(\bar{B}+R_{1}DF(t)E_{2}R_{2})K_{2}Y_{1}x(t-\tau (t))\\ & +Gx_{0}(t)+{\bar{B}}_{1}K_{1,2}\left[\begin{array}{ccc} 0 & 0 & 1 \end{array}\right]x_{0}(t-\tau_{1}) \end{array}$$

with matrices *D* = diag{*D*_1_,*D*_2_,…,*D_N_*}, *E*_1_ = diag{*E*_1,1,_*E*_2,1_,…,*E_N_*_,1_}, *E*_2_ = diag{*E*_1,2_,*E*_2,2_,…,*E_N_*_,2_}, *F*(*t*) = diag{*F*_1_(*t*), *F*_2_(*t*),…,*F_N_*(*t*)},

$$R_{1}={\left[\begin{array}{cccc} 0 & 0 & \cdots & 0\\ 0 & I_{N} & \cdots & 0\\ \cdots & \ddots & \ddots & \cdots \\ 0 & \cdots & 0 & I_{N} \end{array}\right]}_{N\times N},\;\;R_{2}={\left[\begin{array}{cccc} 0 & 0 & \cdots & 0\\ 1 & 0 & \cdots & 0\\ \cdots & \ddots & \ddots & \cdots \\ 0 & \cdots & 1 & 0 \end{array}\right]}_{N\times N}$$

### 3.2. Controller Design

Consider the closed-loop uncertain CACC system in Equation (13) and let *A_k_* = *A* + *DF*(*t*)*E*_1_ + *BK*_1_ + *DF*(*t*)*E*_2_*K*_1_ and $B_{k}=\bar{B}K_{2}Y_{1}+R_{1}DF(t)E_{2}R_{2}K_{2}Y_{1}$ for simplicity. Then the closed-loop system in Equation (13) is equal to

(14) $$\dot{x}(t)=A_{k}x(t)+B_{k}x(t-\tau (t))$$

In what follows, some known lemmas are introduced to derive the robust stability of the uncertain CACC system.

**Lemma** **1.** [31]. *Let P_i_, Q_i_ and R_i_ be any positive-definite symmetric matrices with appropriate dimensions for i = 1 and 2. Then there exists a symmetric matrix M such that*

(15) $$\left[\begin{array}{cc} P_{1}-M & Q_{1}\\ Q_{1}^{T} & R_{1} \end{array}\right]>0,\;\left[\begin{array}{cc} P_{2}+M & Q_{2}\\ Q_{2}^{T} & R_{2} \end{array}\right]>0$$

*if and only if*

(16) $$\left[\begin{array}{ccc} P_{2}+P_{2} & Q_{1} & Q_{2}\\ {}* & R_{1} & 0\\ {}* & * & R_{2} \end{array}\right]>0$$

**Lemma** **2.** [32]. *Given any matrices Q = Q^T^, H and E with appropriate dimensions, the matrix inequality* $Q+HF(t)E+E^{T}F{\left(t\right)}^{T}H^{T}<0$ *holds for the matrix F(t) in inequality Equation (7) if and only if there exists a number ε > 0 such that the matrix inequality* $Q+\varepsilon^{-1}HH^{T}+\varepsilon E^{T}E<0$ *is true*.

Now the robust stability of the uncertain CACC system in Equation (5) of the vehicle platoon subject to the parameter uncertainties in Equation (6) and time-varying delay is summarized as the following theorem.

**Theorem** **1.** *Consider the uncertain closed-loop CACC system in Equation (13). If there exist two positive definite symmetric matrices P∈^3N^^×3N^ and R∈^3N^^×3N^ such that the following nonlinear matrix inequality holds*:

(17) $$\Phi =\left[\begin{array}{ccc} M_{11} & M_{12}^{T} & M_{13}^{T}\\ {}* & -hR^{-1} & 0\\ {}* & * & -hR \end{array}\right]<0$$

*where matrices*$M_{11}=\left[\begin{array}{cc} \vartheta & PB_{k}-N_{1}^{T}+N_{2}\\ {}* & -N_{2}^{T}-N_{2} \end{array}\right]$, *M_12_ = [A_k_ B_k_]h and M_13_ = [N_1_ N_2_]h with ϑ= PA_k_ + A_k_^T^P + N_1_^T^ + N_1_ and some free matrices N_i_∈^3N^^×3N^ for i = 1 and 2, then the CACC system is robust stable*.

**Proof.**  Consider the uncertain closed-loop CACC system in Equation (13). From the Leibniz–Newton formula, it is known that *ϕ*(*t*) = 0 with □

$$\phi (t)=x(t)-x(t-\tau (t))-\int_{t-\tau (t)}^{t}\dot{x}(s)ds$$

Then, for the matrices *N*_1_ and *N*_2_, we have [*x*(*t*)^T^*N*_1_^T^ + *x*(*t*−*τ*(*t*))^T^*N*_2_^T^]*ϕ*(*t*) = 0. Moreover, let *η*_1_(*t*) = [*x*(*t*)^T^ *x*(*t*−*τ*(*t*))^T^]^T^ and *Λ_ij_* = *h*(*S_ij_*− *S_ij_*) with any matrices *S_ij_*∈^3*n*^ ^× 3*n*^ for *i*, *j* = 1, 2. Then *Λ_ij_* = 0 and *η*_1_(*t*)^T^*Λη*_1_(*t*) = 0 with $\Lambda =\left[\begin{array}{cc} \Lambda_{11} & \Lambda_{12}\\ {}* & \Lambda_{22} \end{array}\right]$.

Consider the following Lyapunov–Krasovskii candidate function of the closed-loop system:

(18) $$V(t)=x{\left(t\right)}^{T}Px(t)+\int_{-h}^{0}\int_{t+\theta }^{t}\dot{x}{\left(s\right)}^{T}R\dot{x}(s)dsd\theta$$

Taking the derivative of *V*(*t*) on the time along the trajectories of Equation (13), we have

(19) $$\begin{array}{ll} \dot{V}(t)= & x{\left(t\right)}^{T}(PA_{k}+A_{k}^{T}P)x(t)+2x{\left(t\right)}^{T}PB_{k}x(t-\tau (t))\\ & +\dot{x}{\left(t\right)}^{T}hR\dot{x}(t)-\int_{t-h}^{t}\dot{x}{\left(s\right)}^{T}R\dot{x}(s)ds \end{array}$$

From the changes of the time-delay, we derive that

(20) $$\begin{array}{ll} \dot{V}(t)\leq & x{\left(t\right)}^{T}(PA_{k}+A_{k}^{T}P)x(t)+2x{\left(t\right)}^{T}PB_{k}x(t-\tau (t))\\ & +\dot{x}{\left(t\right)}^{T}hR\dot{x}(t)+\eta_{1}{\left(t\right)}^{T}[\begin{array}{cc} N_{1}^{T} & N_{2}^{T} \end{array}]\phi (t)+\eta_{1}{\left(t\right)}^{T}\Lambda \eta_{1}(t)\\ & -\int_{t-\tau (t)}^{t}\dot{x}{\left(s\right)}^{T}R\dot{x}(s)ds \end{array}$$

which yields

(21) $$\begin{array}{ll} \dot{V}(t)\leq & x{\left(t\right)}^{T}(PA_{k}+A_{k}^{T}P+N_{1}^{T}+h(S_{11}-S_{11}))x(t)\\ & +x{\left(t\right)}^{T}(PB_{k}-N_{1}^{T}+h(S_{12}-S_{12}))x(t-\tau (t))\\ & +x{\left(t-\tau (t)\right)}^{T}(-N_{2}^{T}+h(S_{22}-S_{22}))x(t-\tau (t))\\ & +x{\left(t-\tau (t)\right)}^{T}(B_{k}^{T}P+N_{2}^{T}+h(S_{12}^{T}-S_{12}^{T}))x(t)\\ & +\eta_{1}{\left(t\right)}^{T}{\left[A_{k}^{T}\;B_{k}^{T}\right]}^{T}hR[A_{k}^{}\;B_{k}^{}]\eta_{1}(t)\\ & -\int_{t-\tau (t)}^{t}(\dot{x}{\left(s\right)}^{T}R\dot{x}(s)+x{\left(t\right)}^{T}N_{1}^{T}\dot{x}(s))ds\\ & -\int_{t-\tau (t)}^{t}x{\left(t-\tau (t)\right)}^{T}N_{2}^{T}\dot{x}(s))ds \end{array}$$

Let $\eta_{2}(t,s)={\left[\eta_{1}{\left(t\right)}^{T}\;\dot{x}{\left(s\right)}^{T}\right]}^{T}$. Then, the function *V*(*t*) satisfies that

(22) $$\begin{array}{ll} \dot{V}(t)\leq & x{\left(t\right)}^{T}(PA_{k}+A_{k}^{T}P+N_{1}^{T}+N_{1}+hS_{11})x(t)\\ & +x{\left(t\right)}^{T}(PB_{k}-N_{1}^{T}+N_{2}+hS_{12})x(t-\tau (t))\\ & +x{\left(t-\tau (t)\right)}^{T}(-N_{2}^{T}-N_{2}+hS_{22})x(t-\tau (t))\\ & +x{\left(t-\tau (t)\right)}^{T}(B_{k}^{T}P+N_{2}^{T}-N_{1}+hS_{12}^{T})x(t)\\ & +\eta_{1}{\left(t\right)}^{T}h\Gamma_{1}^{T}R\Gamma_{1}\eta_{1}(t)-\int_{t-\tau (t)}^{t}\eta_{2}{\left(t,s\right)}^{T}\psi \eta_{2}(t,s)ds \end{array}$$

where Γ_1_ = [*A_k_ B_k_*] = *h*^−1^*M*_12_ and $\psi =\left[\begin{array}{ccc} S_{11} & S_{12} & N_{1}^{T}\\ {}* & S_{22} & N_{2}^{T}\\ {}* & * & R \end{array}\right]$. Moreover, the inequality Equation (22) can be re-written as

(23) $$\dot{V}(t)\leq \eta_{1}{\left(t\right)}^{T}(H+h\Gamma_{1}^{T}R\Gamma_{1})\eta_{1}(t)-\int_{t-\tau (t)}^{t}\eta_{2}{\left(t,s\right)}^{T}\psi \eta_{2}(t,s)ds$$

where $H=\left[\begin{array}{cc} H_{11} & H_{12}\\ {}* & H_{22} \end{array}\right]$ with *H*_11_ = *ϑ* + *hS*_11_, *H*_12_ = *PB_k_* − *N*_1_^T^ + *N*_2_ + *hS*_12_ and *H*_22_ = −*N*_2_^T^ − *N*_2_ + *hS*_22_. Clearly, *V*(*t*) monotonically decreases along the trajectories of Equation (13) if *H* + *h*Γ_1_^T^RΓ_1_ < 0 and *ψ* > 0.

To this end, using Shur’s complement [32], it is easy to see that

(24) $$\begin{array}{ll} H+h\Gamma_{1}^{T}R\Gamma_{1}<0 & \Leftrightarrow \left[\begin{array}{cc} H & h\Gamma^{T}\\ {}* & -hR^{-1} \end{array}\right]<0\\ & \Leftrightarrow \left[\begin{array}{cc} -M_{11}-hS & -M_{12}^{T}\\ {}* & hR^{-1} \end{array}\right]>0 \end{array}$$

(25) $$\psi >0\Leftrightarrow \left[\begin{array}{cc} hS & M_{13}^{T}\\ {}* & hR \end{array}\right]>0$$

where $S=\left[\begin{array}{cc} S_{11} & S_{12}\\ {}* & S_{22} \end{array}\right]$. Then applying Lemma 1, it is derived that

(26) $$\left\{\begin{array}{l} H+h\Gamma_{1}^{T}R\Gamma_{1}<0\\ \psi >0 \end{array}\right.\Leftrightarrow \left[\begin{array}{ccc} -M_{11} & -M_{12}^{T} & -M_{13}^{T}\\ {}* & hR^{-1} & 0\\ {}* & * & hR \end{array}\right]>0$$

which is equal to the inequality equation (17). Hence, the condition in Equation (17) yields that *V*(*t*) monotonically decreases along the trajectories of the closed-loop CACC system in Equation (13) and it is a Lyapunov function of the closed-loop CACC system. This establishes the robust stability of the uncertain closed-loop CACC system from the Lyapunov-Krasovskii’s argument [31].

**Remark** **1.** *Theorem 1 presents a sufficient condition to ensure the robust stability of the uncertain closed-loop CACC system in Equation (13). However, the matrix inequality condition inequality Equation (17) is nonlinear, e.g., R^−1^, PA_k_. Hence, it is hard to compute the robust CACC controllers in Equation (9) by the known LMI toolbox. One method is to introduce the linearization method* [31,33] *to compute those CACC controllers via the available LMI toolbox.*

**Theorem** **2.** *Consider the uncertain closed-loop CACC system Equation (13) with some given numbersε_1_ > 0,ε_2_ > 0,λ > 0 andγ > 0. If there exist two symmetric matrices*$0<\bar{P}\in {ℜ}^{3n\times 3n}$*and*$0<\bar{R}\in {ℜ}^{3n\times 3n}$, *and matrices W_1_∈^1^^×3n^ and W_2_∈^1^^×3n^ such that the following LMI holds*:

(27) $$\left[\begin{array}{cccccccc} \Xi_{11} & \Xi_{12} & \Xi_{13} & 0 & \varepsilon_{1}D & \bar{P}E_{1}^{T}+W_{1}^{T}E_{2}^{T} & \varepsilon_{2}R_{1}D & -\lambda \gamma^{-1}W_{2}^{T}R_{2}^{T}E_{2}^{T}\\ {}* & \Xi_{22} & \Xi_{23} & h\bar{R} & 0 & 0 & 0 & \gamma^{-1}W_{2}^{T}R_{2}^{T}E_{2}^{T}\\ {}* & * & -h\bar{R} & 0 & \varepsilon_{1}hD & 0 & \varepsilon_{2}hR_{1}D & 0\\ {}* & * & * & -h\bar{R} & 0 & 0 & 0 & 0\\ {}* & * & * & * & -\varepsilon_{1}I & 0 & 0 & 0\\ {}* & * & * & * & * & -\varepsilon_{1}I & 0 & 0\\ {}* & * & * & * & * & * & -\varepsilon_{2}I & 0\\ {}* & * & * & * & * & * & * & -\varepsilon_{2}I \end{array}\right]<0$$

where $\Xi_{11}=A\bar{P}+\bar{P}A^{T}+BW_{1}+W_{1}^{T}B^{T}-\lambda \gamma^{-1}(\bar{B}W_{2}+W_{2}^{T}{\bar{B}}^{T})$, $\Xi_{12}=\gamma^{-1}\bar{B}W_{2}+\bar{P}+\lambda \gamma^{-1}\bar{P}$, $\Xi_{23}=h\gamma^{-1}W_{2}^{T}{\bar{B}}^{T}$, $\Xi_{13}=h(\bar{P}A^{T}+W_{1}^{T}B^{T}-\lambda \gamma^{-1}W_{2}^{T}{\bar{B}}^{T})$, and $\Xi_{22}=-2\gamma^{-1}\bar{P}$, then the uncertain closed-loop CACC system is robust stable with the CACC gains $K_{1}=W_{1}{\bar{P}}^{-1}$ and $K_{2}=W_{2}{\bar{P}}^{-1}Y_{1}^{-1}$.

**Proof.**  Let $W=\left[\begin{array}{cc} P & 0\\ N_{1} & N_{2} \end{array}\right]$ and $\bar{A}=\left[\begin{array}{cc} A_{k} & B_{k}\\ I & -I \end{array}\right]$. Then the matrices *M*_11_ and *M*_13_ in the nonlinear matrix inequality Equation (17) can be expressed as □

(28) $$M_{11}=W^{T}\bar{A}+{\bar{A}}^{T}W,\;M_{13}^{T}=hW^{T}{\left[\begin{array}{cc} 0 & 1 \end{array}\right]}^{T}$$

Let $N_{1}=\lambda P,N_{2}=\gamma P$ with γ≠0 and then *W* is reversible

(29) $$W^{-1}=\left[\begin{array}{cc} P^{-1} & 0\\ -\lambda \gamma^{-1}P^{-1} & \gamma^{-1}P^{-1} \end{array}\right]$$

Define a matrix *T* as

(30) $$T=\left[\begin{array}{ccc} W^{-1} & 0 & 0\\ {}* & I & 0\\ {}* & * & R^{-1} \end{array}\right]$$

Multiply the matrix Φ in inequality Equation (17) left by *T*^T^ and right by *T* simultaneously as

(31) $$T^{Τ}\Phi T=\left[\begin{array}{ccc} {\tilde{M}}_{11} & W^{-Τ}M_{12}^{T} & \Pi_{1}^{Τ}\\ {}* & -hR^{-1} & 0\\ {}* & * & -hR^{-1} \end{array}\right]$$

where ${\tilde{M}}_{11}=\bar{A}W^{-1}+W^{-T}{\bar{A}}^{T}$ and $\Pi_{1}=\left[\begin{array}{cc} 0 & hR^{-1} \end{array}\right]$.

Let $\bar{P}=P^{-1}$,$\bar{R}=R^{-1}$,$W_{1}=K_{1}\bar{P}$, and $W_{2}=K_{2}Y_{2}\bar{P}$. Substituting *W*, *A_k_* = *A* + *DF(t)E*_1_ *+ BK*_1_ + *DF(t)E*_2_*K*_1_, $\bar{A}$, and $B_{k}=\bar{B}K_{2}Y_{1}+R_{1}DF(t)E_{2}R_{2}K_{2}Y_{1}$ into Equation (31), we have that

(32) $$T^{Τ}\Phi T=\Psi_{1}+\Psi_{2}$$

with

$$\Psi_{1}=\left[\begin{array}{cccc} \Xi_{11} & \Xi_{12} & \Xi_{13} & 0\\ {}* & \Xi_{22} & \Xi_{23} & h\bar{R}\\ {}* & * & -h\bar{R} & 0\\ {}* & * & * & -h\bar{R} \end{array}\right],\;\Psi_{2}=\left[\begin{array}{cccc} \Sigma_{11} & \Sigma_{12} & \Sigma_{13} & 0\\ {}* & 0 & \Sigma_{23} & 0\\ {}* & * & 0 & 0\\ {}* & * & * & 0 \end{array}\right]$$

where

$\Xi_{11}=A\bar{P}+\bar{P}A^{T}+BW_{1}+W_{1}^{T}B^{T}-\lambda \gamma^{-1}(\bar{B}W_{2}+W_{2}^{T}{\bar{B}}^{T})$,
$\Xi_{12}=\gamma^{-1}\bar{B}W_{2}+\bar{P}+\lambda \gamma^{-1}\bar{P}$,
$\Xi_{23}=h\gamma^{-1}W_{2}^{T}{\bar{B}}^{T}$,
$\Xi_{13}=h(\bar{P}A^{T}+W_{1}^{T}B^{T}-\lambda \gamma^{-1}W_{2}^{T}{\bar{B}}^{T})$,
$\Xi_{22}=-2\gamma^{-1}\bar{P}$, $\Sigma_{12}=\gamma^{-1}R_{1}DF(t)E_{2}R_{2}W_{2}$,
$\Sigma_{11}=DF(t)E_{1}\bar{P}+\bar{P}E_{1}^{T}F{\left(t\right)}^{T}D^{T}+DF(t)E_{2}W_{1}+W_{1}^{T}E_{2}^{T}F{\left(t\right)}^{T}D^{T}-\lambda \gamma^{-1}(R_{1}DF(t)E_{2}R_{2}W_{2}+W_{2}^{T}R_{2}^{T}E_{2}^{T}F{\left(t\right)}^{T}D^{T}R_{1}^{T})$,
$\Sigma_{13}=h(\bar{P}E_{1}^{T}F{\left(t\right)}^{T}D^{T}+W_{1}^{T}E_{2}^{T}F{\left(t\right)}^{T}D^{T}-\lambda \gamma^{-1}W_{2}^{T}R_{2}^{T}E_{2}^{T}F{\left(t\right)}^{T}D^{T}R_{1}^{T})$,
$\Sigma_{23}=h\gamma^{-1}W_{2}^{T}R_{2}^{T}E_{2}^{T}F{\left(t\right)}^{T}D^{T}R_{1}^{T}$.

Moreover, let $H1={\left[\begin{array}{cccc} D^{T} & 0 & hD^{T} & 0 \end{array}\right]}^{T}$, $E1=\left[\begin{array}{cccc} E_{1}\bar{P}+E_{2}W_{1} & 0 & 0 & 0 \end{array}\right]$, $H2={\left[(R_{1}\begin{array}{cccc} D{)}^{T} & 0 & h{\left(R_{1}D\right)}^{T} & 0 \end{array}\right]}^{T}$, and $E2=\left[\begin{array}{cccc} -\lambda \gamma^{-1}E_{2}R_{2}W_{2} & \gamma^{-1}E_{2}R_{2}W_{2} & 0 & 0 \end{array}\right]$. Substituting the matrices *H*1, *H*2, *E*1 and *E*2 into Ψ_2_, it is obtained that

(33) $$T^{Τ}\Phi T=\Psi_{1}+H1F(t)E1+E1^{Τ}F{\left(t\right)}^{Τ}H1^{Τ}+H2F(t)E2+E2^{Τ}F{\left(t\right)}^{Τ}H2^{Τ}$$

From Lemma 2, it is derived that

(34) $$T^{T}\Phi T<0\Leftrightarrow \Psi_{1}+\varepsilon_{1}H1H1^{T}+\varepsilon_{1}^{-1}E1E1^{T}+\varepsilon_{2}H2H2^{T}+\varepsilon_{2}^{-1}E2E2^{T}<0$$

for any numbers *ε*_1_, *ε*_2_ > 0. Using Shur’s complement [32], the inequality equation (34) is equivalent to the following LMI:

(35) $$\left[\begin{array}{ccccc} \Psi_{1} & \varepsilon_{1}H1 & E1^{T} & \varepsilon_{2}H2 & E2\\ {}* & -\varepsilon_{1}I & 0 & 0 & 0\\ {}* & * & -\varepsilon_{1}I & 0 & 0\\ {}* & * & * & -\varepsilon_{2}I & 0\\ {}* & * & * & * & -\varepsilon_{2}I \end{array}\right]<0$$

which equals Equation (27) with $K_{1}=W_{1}{\bar{P}}^{-1}$ and $K_{2}=W_{2}{\bar{P}}^{-1}Y_{1}^{-1}$. Then from Theorem 1, the uncertain closed-loop system in Equation (13) is robust stable. This completes the proof of this theorem.

Note that being different from the matrix inequality equation(17) in Theorem 1, the matrix inequality Equation (27) in Theorem 2 is linear with respect to the matrices $\bar{P}$, $\bar{R}$, *W*_1_ and *W*_2_. Hence, these matrices can be obtained by solving the feasibility problem of Equation (27) with the solver ‘feasp’ in the LMI toolbox [32].

## 4. String Stability Analysis

Although the uncertain closed-loop CACC system in Equation (12) or Equation (13) guarantees the zero steady-state spacing error for each vehicle in the platoon, it gives no specific restrictions on the transient spacing errors and string stability. In order to meet the string stability requirement, i.e., the transient spacing errors are not amplified downstream along the platoon, the CACC system needs to derive the conditions on transient spacing errors of the whole vehicle platoon.

Considering the desired safe spacing in Equation (4), we have the Laplace transform of the spacing error and the relative velocity of adjacent vehicles, respectively

(36) $$\delta_{i}(s)=\left[a_{i-1}(s)-a_{i}(s)\right]/s^{2}-T_{i}a_{i}(s)/s,\;\Delta v_{i}(s)=\left[a_{i-1}(s)-a_{i}(s)\right]/s$$

Substituting Equation (9) and Equation (36) into Equation (3) with some algebra operations, it is obtained that

(37) $$G_{i}(s)=\frac{a_{i}(s)}{a_{i-1}(s)}=\frac{k_{i,1}+k_{i,2}s+k_{i,4}s^{2}e^{-\tau_{i}s}}{s^{3}\varsigma_{i}+(1-k_{i,3})s^{2}+(T_{i}k_{i,1}+k_{i,2})s+k_{i,1}}$$

where the controller gain *K_i_* = [*k_i_*_,1_, *k_i_*_,2_, *k_i_*_,3_, *k_i_*_,4_] of each vehicle *i* is determined as Theorem 2. Then we have the following robust string stability of the vehicle platoon with the CACC system Equation (13).

**Theorem** **3.** *Consider the vehicle platoon with the CACC system in Equation (13). The transfer function G_i_(s) in Equation (37) satisfies that ‖G_i_(jw)‖≤ 1 for any w≥ 0 if its parameters satisfy the following inequalities*

(38) $$1+k_{i,3}^{2}-k_{i,4}^{2}-2k_{i,3}-2\varsigma_{i}T_{i}k_{i,1}-2\varsigma_{i}k_{i,2}-3k_{i,4}k_{i,1}\geq 0$$

(39) $$2k_{i,1}k_{i,3}+T_{i}{}^{2}k_{i,1}^{2}+2T_{i}k_{i,1}k_{i,2}-2k_{i,1}+2k_{i,4}k_{i,1}\geq 0$$

*for i = 1,..,N, i.e., the uncertain vehicle platoon with the CACC system has robust string stability*.

**Proof.**  Let *s* = j*w* and substitute it into *G_i_*(*s*). From the Euler formula on *e*^−^*^τis^*, we obtain that □

(40) $$\left\|G_{i}(jw)\right\|=\left\|\frac{k_{i,1}+jwk_{i,2}-k_{i,4}w^{2}(\cos(w\tau_{i})-j\sin(w\tau_{i}))}{-jw^{3}\varsigma_{i}-(1-k_{i,3})w^{2}+j(T_{i}k_{i,1}+k_{i,2})w+k_{i,1}}\right\|=\sqrt{\frac{\alpha (w)}{\alpha (w)+\beta (w)}}$$

where

$$\begin{array}{l} \alpha (w)={\left[k_{i,1}-k_{i,4}w^{2}\cos(\tau_{i}w)\right]}^{2}+{\left[k_{i,2}w+k_{i,4}w^{2}\sin(\tau_{i}w)\right]}^{2},\\ \beta (w)=[\varsigma_{i}^{2}w^{4}+(1-2\varsigma_{i}T_{i}k_{i,1}-2\varsigma_{i}k_{i,2}-2k_{i,3}+k_{i,3}^{2}-k_{i,4}^{2})w^{2}-2k_{i,2}k_{i,4}\sin(\tau_{i}w)w\\ \;\;\;+2k_{i,1}k_{i,4}\cos(\tau_{i}w)-2k_{i,1}+2T_{i}k_{i,1}k_{i,2}+2k_{i,1}k_{i,3}+T_{i}{}^{2}k_{i,1}^{2}]w^{2}. \end{array}$$

Clearly, *α*(*w*) > 0 for any *w* > 0. Hence, it is true that ‖*G_i_*(j*w*)‖=‖*a_i_*(j*w*)/*a_i_*_−1_(j*w*)‖ ≤ 1 for any *w* > 0 and *I* = 1,…,*N*, if *β*(*w*) > 0 for any *w* > 0. Equally, the following inequality

(41) $$\begin{array}{l} w^{4}\varsigma_{i}^{2}+[1+k_{i,3}^{2}-k_{i,4}^{2}-2k_{i,3}-2\varsigma_{i}T_{i}k_{i,1}-2\varsigma_{i}k_{i,2}]w^{2}-2k_{i,2}k_{i,4}\sin(\tau_{i}w)w\\ +2k_{i,4}k_{i,1}\cos(\tau_{i}w)+2k_{i,1}k_{i,3}+T_{i}{}^{2}k_{i,1}^{2}+2T_{i}k_{i,1}k_{i,2}-2k_{i,1}\geq 0 \end{array}$$

holds for any *w* > 0. Using Taylor series on sin(*τ_i_w*) and cos(*τ_i_w*), we have

(42) $$\sin(\tau_{i}w)\approx \tau_{i}w-{\left(\tau_{i}w\right)}^{3}/3!,\;\cos(\tau_{i}w)\approx 1-{\left(\tau_{i}w\right)}^{2}/2!$$

Substituting Equation (42) into Equation (41), it is derived that

(43) $$\begin{array}{l} (\varsigma_{i}^{2}+\frac{k_{i,2}k_{i,4}\tau_{i}^{3}}{3})w^{4}+[1+k_{i,3}^{2}-k_{i,4}^{2}-2k_{i,3}-2\varsigma_{i}T_{i}k_{i,1}-2\varsigma_{i}k_{i,2}-2k_{i,4}k_{i,1}-k_{i,4}k_{i,1}\tau_{i}^{2}]w^{2}\\ +2k_{i,1}k_{i,3}+T_{i}^{2}k_{i,1}^{2}+2T_{i}k_{i,1}k_{i,2}-2k_{i,1}+2k_{i,4}k_{i,1}\geq 0 \end{array}$$

Then noticing the conditions Equation (38) and Equation (39) with $\varsigma_{i}>0,k_{i,1},k_{i,2},k_{i,4}>0,k_{i,3}<0,\tau_{i}\leq 1$, we obtain from Equation (43) that ‖*G_i_*(j*w*)‖ ≤ 1 for any *w* ≥ 0 and *i* = 1,…,*N*. Hence, the robust string stability of the uncertain vehicle platoon with the CACC system in Equation (13) is ensured.

## 5. Numerical Experiments

In this section, a group of seven CAVs is used to evaluate the robust stability and robust string stability performances of the proposed CACC method for the vehicle platoon system. The vehicle platoon considered here runs in a single lane road with a varying speed traffic scenario consisting of quickly accelerating case, normal cruise cases with high and low speeds and fast decelerating case. The actual traffic scenarios are usually combinations of these basic cases. In this simulation, the CACC controller implemented in each car in the vehicle platoon is calculated by LMI software package in MATLAB 2014a. The control goal of this study is to avoid collision among cars in the vehicle platoon and to keep the desired safety spacing with common speeds in the presence of system uncertainties and varying communication delay, namely, to achieve the robust stability and robust string stability performances for the uncertain CACC system with varying delay. 

In the study, the nominal values of the vehicles’ parameters are selected empirically as following: length of vehicles *Li* = 2 m, time constants *ς_I_* = 0.2 s, time gap *Ti* = 1.05 s and desired safety spacing *D_i_* = 8 m for *i* = 1,…,6. The upper bound of varying communication delay of the CACC system considered here is picked as *h* = 1.0 s [19,30]. In simulation, the uncertainties Δ*A_i_* and Δ*B_i_* of Equation (5) are caused by the uncertain time gap of each car with the change range of [0, 0.2], i.e., the real value of time gap of each car in the vehicle platoon is selected stochastically within the range of [0.2, 0.4]. Then, the uncertainties Δ*A_i_* and Δ*B_i_* satisfy that $\left[\begin{array}{cc} \Delta A_{i} & \Delta B_{i} \end{array}\right]=D_{i}F_{i}(t)\left[\begin{array}{cc} E_{i,1} & E_{i,2} \end{array}\right]$ and $F_{i}{}^{T}(t)F_{i}(t)\leq I$ with the constant matrices *D_i_* = [0 0 0; 0 0 0; 0 0 1], *E_i_*_,1_=[0 0 0; 0 0 0; 0 0 −1.67] and *E_i_*_,2_ = [0; 0; 1.67] for each vehicle *i* = 1,…,6. As the traffic messages of the seven vehicles and the acceleration of front vehicles are transmitted by wireless V2V channels, in simulation, the varying communication delay, *τ_i_*, of the vehicle platoon are produced by the MatLab function ‘rand’ with the upper bound *h*. Moreover, in order to compute the CACC gains *K_i_*, we select *λ* = −0.2, *γ* = 0.037, *ε*_1_ = 0.05 and *ε*_2_ = 0.01 by the empirically trial and error method. Then by solving the LMIs in Equation (27) with the solver ‘feasp’ in the LMI toolbox [32], we have the gains of the CACC controllers of the vehicle platoon, which are shown as Table 1. 

**Figure 2:**
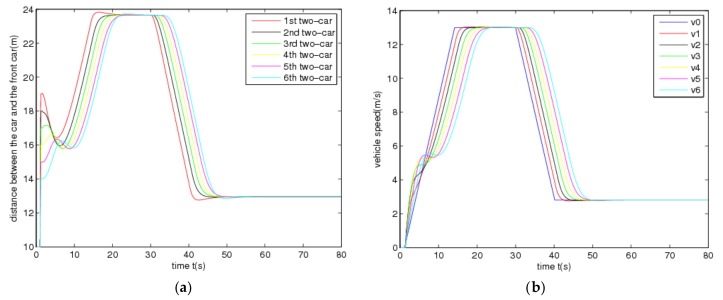

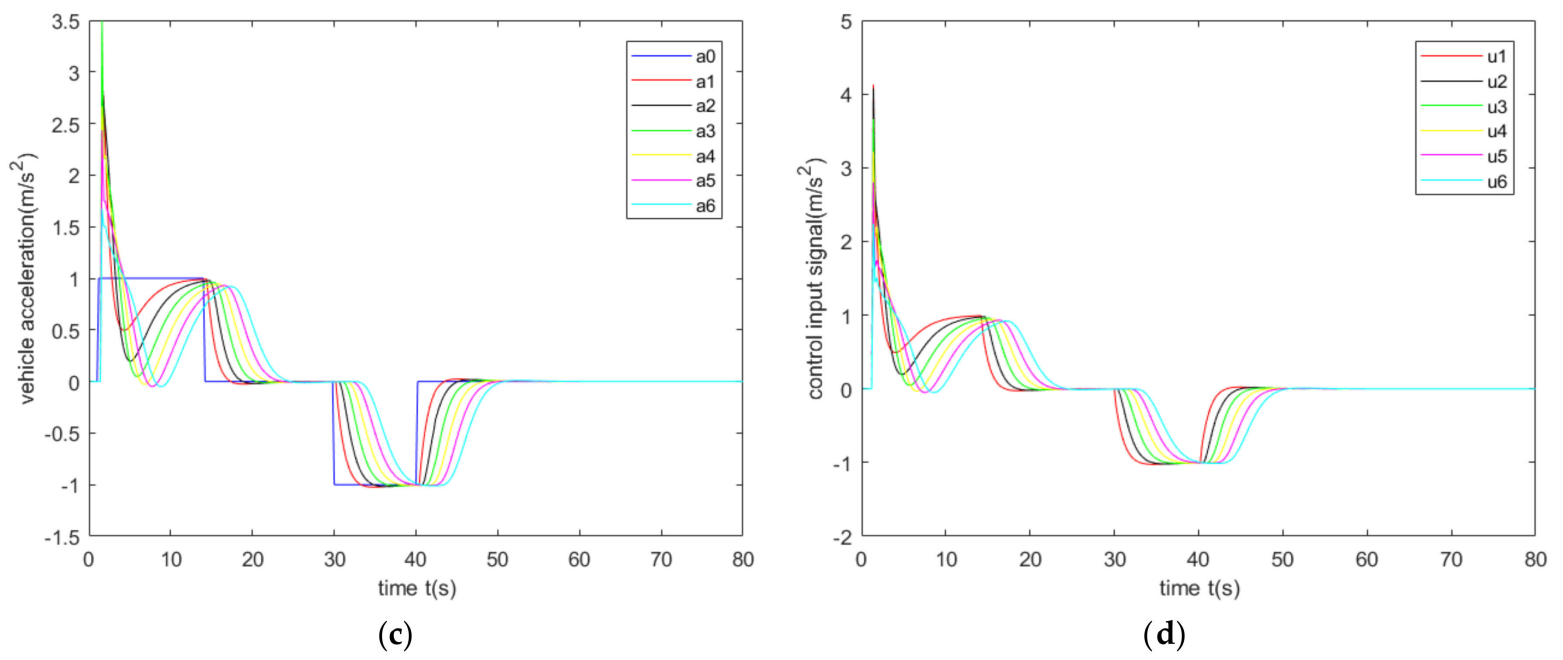
Time evolutions of states and control of the vehicle platoon: (**a**) Actual spacing, (**b**) Velocity, (**c**) Acceleration, (**d**) Control signal.

In the simulation study, the initial spacing errors *e_i_*(0) of the vehicle platoon are set as *e*_1_(0) = 9 m, *e*_2_(0) = 8 m, *e*_3_(0) = 7 m, *e*_4_(0) = 6 m, *e*_5_(0) = 5 m and *e*_6_(0) = 4 m, and all cars of the vehicle platoon stop at initial time *t*=0. To begin with, the leading vehicle accelerates at 1 m/s^2^ from 1 to 13 s and then cruises at the constant speed 13 m/s for lasting 8 secs. At time 31 s, due to the emerging traffic scenarios, e.g., front jamming, cars suddenly cutting-in from adjacent lanes, etc., the leading vehicle decelerates fast until the velocity gets to 2.9 m/s at the time 41 s and then cruises at the low speed. These driving behaviors are very common in real traffic scenarios, especially in urban environments. Under these traffic scenarios, Figure 2 shows the time evolutions of all vehicles controlled by the designed CACC controllers with the gains in Table 1, where the subplot (a) pictures the actual inter-vehicle distance (spacing) profiles between the adjacent vehicles and the subplots (b)–(d) picture the time evolutions of the vehicle velocity, acceleration and computed control signals of the host vehicles, respectively. Note that in simulation, the varying time-delay of the communication network is produced by a stochastic signal satisfying the range of [0, *h*] with *h =* 1.0 s. 

**Figure 3:**
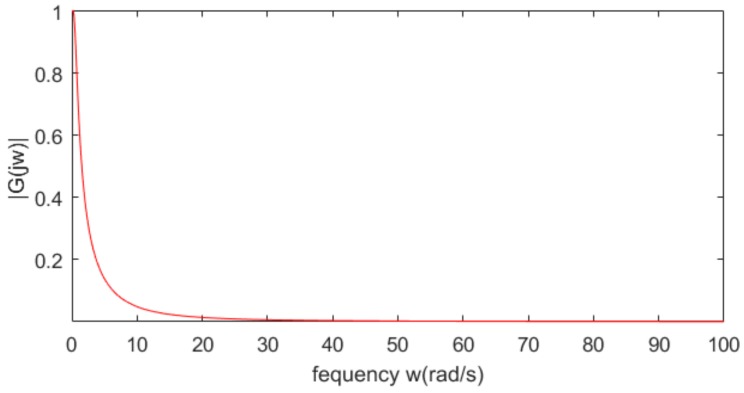
Frequency response for any *w* ≥ 0.

From Figure 2, it is observed that the trajectories of all vehicles can quickly converge in the face of the uncertainties and varying time-delay induced by the communication transmission. In other words, each car has the ability to track its immediately preceding one and maintain the inter-vehicle distance at the desired safety spacing as well as ensuring common speeds of all cars for the vehicle platoon subject to uncertainties and varying delay under the varying speed traffic scenario. This suggests that the vehicle platoon with the designed CACC controllers is robust stable in the presence of the uncertainties and varying communication delay. Note that at the starting, each following vehicle has a fast, transient behavior of acceleration and control input in order to quickly response the behaviors of the leading vehicle. Due to different uncertainties, varying time-delay and initial state of each vehicle, the transient behaviors of all vehicle are different accordingly. Moreover, Figure 3 shows that the frequency response of the acceleration transfer function, *G_i_*(j*w*), on the adjacent vehicles in the vehicle platoon with the CACC system. From Figure 3, it is observed that the frequency response of *G_i_*(j*w*) satisfies that ‖*G_i_*(j*w*)‖ ≤ 1 for any *w* ≥ 0, *i* = 1,…,6 in the presence of the uncertainties and varying communication delay. As a result, it is known from Definition 2 and Figure 2 and Figure 3 that the vehicle platoon with the CACC system has robust string stability for the uncertainties and varying time-delay of range [0, *h*]. This implies that the vehicle platoon with the designed CACC controllers has ability to attenuate spacing errors and velocity fluctuations resulted from the cars in front even in the presence of the uncertainties and varying communication delay. The robust stability and robust string stability performances of the CACC system are beneficial to improve and keep smooth traffic flow of roads. These results illustrate the effectiveness of the proposed robust CACC method for vehicle platoons subject to uncertainties and varying communication delay.

**Figure 4:**
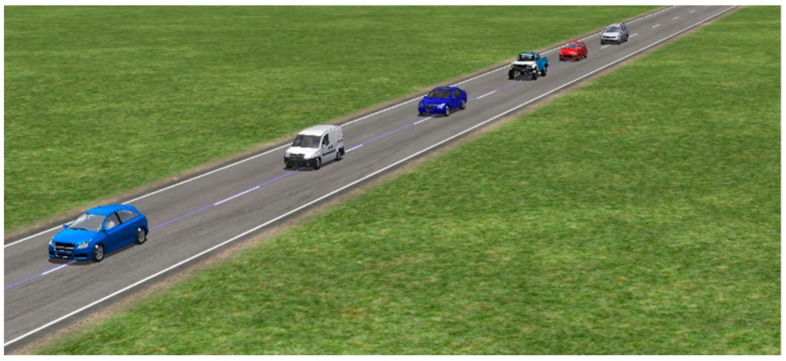
The six-vehicle platoon with CACC in CarSim.

In order to further demonstrate the effectiveness of the presented CACC method, we use the CarSim2016.1 software to evaluate the stability performance of the control method. The software is a high-fidelity simulation environment with an interface for MATLAB/Simulink. In this experiment, six heterogeneous vehicles are used to make up a vehicle platoon which is shown in Figure 4. The vehicle types of the six vehicles are selected as C-Class Hatchback, European Van, F-Class Sedan, Off-Road Pickup, B-Class Hatchback, and D-Class Minivan, respectively. These different cars will be used to illustrate the applicability of the proposed CACC method to heterogeneous vehicle platoon. The velocity and acceleration of the leading car and simulation parameters are selected to be the same as the aforementioned MATLAB numerical experiment. The remaining parameters of these vehicles, e.g., the mass, transmission system and other parameters are selected to be the default values of the software.

**Figure 5:**
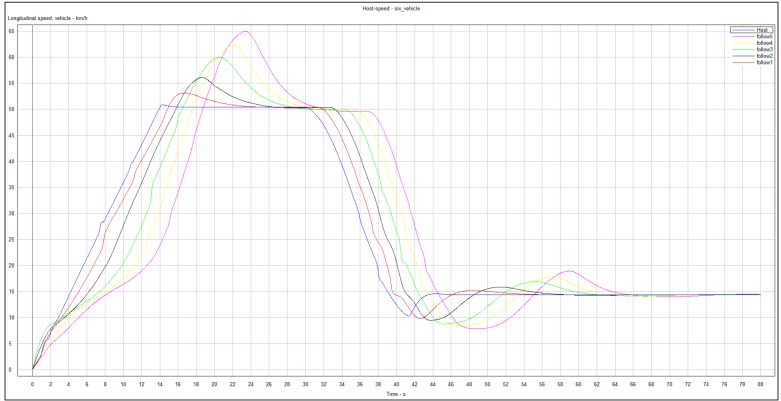
Velocity profiles of the six vehicles.

**Figure 6:**
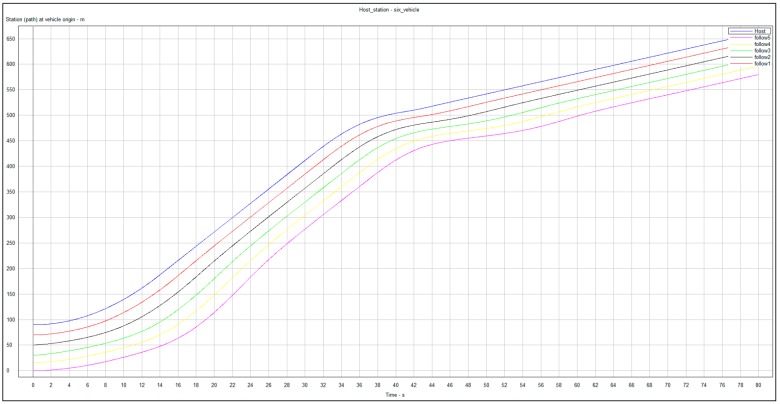
Position profiles of the six vehicles.

In this co-simulation experiment of CarSim and Simulink, the CarSim software outputs the position, velocity and acceleration of each vehicle to Simulink at each time. Then, with the default parameters of the vehicular powertrain system and braking system, the throttle opening, or the main cylinder pressure output of each vehicle is computed by the input of CarSim and the controller in Simulink. The readers refer [34] for detailed co-simulation setup of CarSim and Simulink. Figure 5 and Figure 6 show the time profiles of velocity and position of the six vehicles, respectively. From Figure 5 and Figure 6, it can be seen that the vehicle platoon with the designed CACC controllers is robust stable and robust string stable in the presence of the uncertainties and varying time-delay. These results are the similar to those of the aforesaid MatLab simulation experiment, which further verify the robustness performance of the proposed CACC approach for vehicle platoons subject to uncertainties and varying communication delay. 

From the aforesaid simulation results and discussions, it is known that the robust time-delay feedback CACC method is an effective strategy employed to achieve the goal of CACC for the vehicle platoon systems subject to uncertainties and varying communication delay. Therefore, these simulation results demonstrate the robust stability and robust string stability performances of the robust CACC method proposed in this paper. 

## 6. Conclusions

In this paper, we presented a new robust cooperative adaptive cruise control method for vehicle platoons subject to parametric uncertainties and varying communication delay. A set of robust delay feedback CACC controllers was designed for the uncertain vehicle platoon with varying communication delay and was computed by solving some linear matrix inequalities. Moreover, the LMI conditions were established to guarantee the robust stability and robust string stability properties of the vehicle platoon in the presence of the uncertainties and varying time-delay. The results of simulation experiments demonstrated the effectiveness of the robust CACC method presented in this paper and evaluated the robust stability and robust string stability properties of the vehicle platoon subject to uncertainties and varying communication delay. 

Since there generally exist nonlinearities and uncertainties in real vehicles, some interesting directions of future work include the investigation on nonlinear CACC methods and robust (string) stability of nonlinear vehicle platoons subject to varying time-delay induced by heterogeneous communication and vehicles’ actuators.

## Figures and Tables

**Table 1:** The computed CACC gains of each vehicle.

Car *i*	*k_i_*_,1_	*k_i_*_,2_	*k_i_*_,3_	*k_i_*_,4_
1	0.6368	1.7098	−1.0715	1.60 × 10^−4^
2	0.7140	1.7821	−0.9418	1.60 × 10^−4^
3	0.7112	1.6802	−0.8386	1.64 × 10^−4^
4	0.7163	1.6595	−0.8426	4.45 × 10^−4^
5	0.7479	1.7292	−0.9590	1.21 × 10^−3^
6	0.7753	1.5510	−1.0210	2.70 × 10^−3^
